# Nutrient-Sensitive Epigenetic Modifiers as Candidate Biomarkers of Metabolic Dysfunction in Obesity: A Nutrigenomic Review

**DOI:** 10.3390/ijms27104372

**Published:** 2026-05-14

**Authors:** Diana Rodríguez-Vera, Manuel Abraham Gómez-Martínez, Mildred Valeria Herrera-Picazo, Liliana Anguiano Robledo, Cecilia Tufiño, Claudia C. Bustamante-Tenorio, Marvin A. Soriano-Ursúa, Ángel Morales-González, Eduardo Osiris Madrigal-Santillán, Arely Vergara-Castañeda, José Antonio Morales-González

**Affiliations:** 1 Section of Postgraduate Studies and Research, Higher School of Medicine, Instituto Politécnico Nacional, Plan de San Luis y Díaz Mirón s/n, Mexico City 11340, Mexico; nutriologadianavera@gmail.com (D.R.-V.); msoriano@ipn.mx (M.A.S.-U.); 2 Béke Specialized Nutrition Clinic, Texcoco 56130, Mexico; nutclaudiabeke@gmail.com; 3 Public Health Department, Faculty of Medicine, Universidad Nacional Autónoma de México, Escolar 411A, Copilco Universidad, Coyoacán, Mexico City 04360, Mexico; mgomez@facmed.unam.mx; 4 Laboratorio de Medicina de Conservación, Escuela Superior de Medicina, Instituto Politécnico Nacional, Plan de San Luis y Díaz Mirón s/n, Mexico City 11340, Mexico; mildred.herrera13@gmail.com (M.V.H.-P.); eomsmx@yahoo.com.mx (E.O.M.-S.); 5 Molecular Pharmacology Laboratory, Escuela Superior de Medicina, Instituto Politécnico Nacional, Mexico City 07738, Mexico; languianorobledo@yahoo.com.mx; 6 Pregnancy Pharmacology Laboratory, Escuela Superior de Medicina, Instituto Politécnico Nacional, Plan de San Luis y Díaz Mirón s/n, Mexico City 11340, Mexico; cecil.ctm@gmail.com; 7 Escuela Superior de Computo, Instituto Politécnico Nacional, “A. López Mateos” Professional Unit, Mexico City 07738, Mexico; anmorales@ipn.mx; 8 Promotion and Education for Health and Food Research Group, Chemical Sciences School, Universidad La Salle, Benjamin Franklin No. 47 Col. Condesa, Mexico City 06140, Mexico

**Keywords:** nutrigenomics, obesity, metabolic disorder, biomarkers

## Abstract

Obesity is a complex metabolic disorder resulting from interactions among genetic, environmental, and dietary factors. Traditional clinical markers may provide limited insight into the biochemical mechanisms that link nutrition and metabolic dysfunction; in this context, the epigenetic mechanisms through which nutrients modulate gene expression are central to understanding metabolic homeostasis. This review summarizes the published evidence on nutrient-driven epigenetic processes in obesity, focusing on DNA methyl donors, such as folate, vitamin B12, choline, betaine, serine, and methionine, and their effects on methylation and DNA methyltransferase activity. Metabolites such as acetyl-CoA, NAD^+^, and short-chain fatty acids (SCFAs) can also influence histone modifications, while diet-responsive microRNAs can regulate networks involved in adipogenesis, lipid metabolism, inflammation, and insulin signaling. Recent studies have identified epigenetic signatures associated with adiposity and metabolic traits, many of which are linked to the risk of cardiometabolic disease. This review is structured around the concept that nutrient-sensitive epigenetic mechanisms act as candidate biomarkers, linking dietary exposure to metabolic dysfunction. Recent evidence supports the idea that nutrient–epigenetic variation could complement traditional metabolic evaluations by offering mechanistic insight and translational direction. These findings suggest that nutrient-sensitive epigenetic mechanisms are biologically plausible candidate biomarker layers; however, their clinical implementation is currently limited by issues including tissue specificity, reproducibility, and the need for prospective validation.

## 1. Introduction

Obesity and related metabolic disorders remain major global health challenges, as people with similar body measurements often have very different metabolic risks, inflammatory profiles, and therapeutic responses. Traditional measures such as body mass index (BMI), which have long been used to assess these conditions, offer little insight into the molecular processes that distinguish healthy from dysfunctional metabolism. This limitation highlights the need for molecular biomarkers that capture tissue-level regulatory events, improve mechanistic interpretation, and support the early identification and personalized management of these conditions [1].

Gene regulation plays a key role in maintaining metabolic homeostasis by integrating genetic and environmental factors, with diet being a central influence. Epigenetic mechanisms, such as DNA methylation, histone modifications, and non-coding RNA, modify chromatin and gene expression without altering the DNA sequence [2]. These processes enable adaptation to changes in nutrients and record both short- and long-term metabolic experiences. Furthermore, nutritional imprinting intersects with age-related epigenetic drift, which can potentially increase susceptibility to metabolic imbalance [3].

Notably, the diet can influence epigenetic regulation not only through caloric excess or restriction, but also through the quality and type of nutrients consumed [4]. Nutrients and bioactive food components act as substrates, cofactors, or regulators of epigenetic enzymes, supporting the concept of nutrient-sensitive epigenetic regulation. Through these mechanisms, dietary patterns and specific nutrients can modulate the gene programs involved in adipogenesis, mitochondrial function, lipid metabolism, inflammation, and insulin signaling [5]. These regulatory processes occur in metabolic tissues such as adipose tissue, the liver, muscles, and the intestinal epithelium, where nutrient sensing is closely linked to metabolic and immune functions [6].

One-carbon metabolism also depends on the balance between S-adenosylmethionine (SAM) and S-adenosylhomocysteine (SAH), which can alter DNA methylation patterns at genes essential for energy metabolism, adipogenesis, and insulin sensitivity when disrupted. Thus, one-carbon metabolism is a key nutrient-sensitive hub in metabolic regulation [7]. In addition to DNA methylation, it is important to note that nutrient-dependent histone modifications can influence chromatin behavior, while cellular energy status and metabolite availability can regulate histone acetylation and deacetylation [8]. This occurs via pathways involving acetyl-CoA, NAD^+^, histone acetyltransferases (HATs), histone deacetylases (HDACs), and sirtuins (SIRTs). Furthermore, SCFAs generated via dietary fiber fermentation, particularly butyrate, link gut bacteria to host epigenetic regulation by inhibiting HDACs [9]. Diet-regulated microRNAs also exert control over signaling pathways involved in lipid metabolism, inflammation, and insulin function [10]. Taken together, these mechanisms illustrate the complexity of epigenetic regulation in response to diverse dietary signals.

Human studies have demonstrated the importance of various mechanisms in obesity and metabolic diseases; for example, epigenome-wide association studies (EWAS) have consistently identified DNA methylation patterns associated with BMI, central adiposity, insulin resistance, and dyslipidemia throughout varied populations [11]. Multi-omics integration provides an organized framework for contextualizing epigenetic findings within broader metabolic networks. Through aligning methylation signatures with transcriptomic and metabolomic perturbations, nutrigenomic approaches can clarify mechanistic pathways linking dietary exposures to metabolic phenotypes. Such integrative strategies strengthen biological plausibility and improve the interpretability of candidate epigenetic biomarkers for translation. Together, these human data highlight the importance of nutrient-sensitive epigenetic modifications in disease risk.

It should be noted that reverse causality, smoking, medication exposure, inflammation, and effects on cell composition can influence methylation estimates. Furthermore, nutritional and lifestyle interventions have demonstrated that epigenetic marks are dynamic and modifiable, serving as sensitive indicators of environmental and metabolic changes [12]. As such, reproducibility alone should not be interpreted as proof of causality or immediate clinical utility [13].

Unlike previous descriptive reviews, this manuscript emphasizes the importance of epigenetic modifiers as both mechanistic intermediates and candidate biomarker layers that connect dietary exposures to clinically interpretable metabolic phenotypes. The text is organized in a logical progression from mechanistic to human evidence, then to translational evidence. It moves from nutrient-dependent chromatin regulation to population-level epigenetic signatures, multi-omics integration, and public health relevance.

Accordingly, the three aims of the study are as follows: first, to summarize the major nutrient-responsive epigenetic mechanisms implicated in obesity; second, to examine human evidence supporting the proposed biomarker candidates; and third, to outline the opportunities and limitations of applying epigenetic information to precision nutrition and public health strategies.

Regardless, human evidence remains challenging to obtain. Most EWAS have been conducted in accessible tissues such as blood, where obesity-related biology is distributed across adipose tissue depots, the liver, skeletal muscle, and neuroendocrine circuits.

Therefore, the following section examines the basis on which nutrients may influence chromatin regulation, before turning to tissue-specific signatures, human evidence, and translational implications.

## 2. Mechanistic Basis: Nutrients as Epigenetic Modulators

### 2.1. One-Carbon Metabolism and DNA Methylation

One-carbon metabolism is an essential biochemical network integrating the folate and methionine cycles to support critical cellular functions, including nucleotide synthesis, maintenance of the redox state, and overall methylation capacity [14]. This network depends on several essential nutrients, such as folate, vitamin B12, choline, betaine, methionine, and vitamin B6. All of these nutrients are required for the production and utilization of SAM, the primary methyl donor for the methylation of DNA, RNA, proteins, and histones [15]. Meanwhile, SAH can accumulate and inhibit methyltransferases, thereby reducing cellular methylation potential. The SAM/SAH ratio acts as a key regulator linking nutrition and epigenetic regulation [16].

Accordingly, folate-dependent one-carbon flux, with vitamin B12-dependent remethylation and choline/betaine-mediated backup pathways, collectively determine methyl group availability for SAM synthesis and, therefore, set the methylation potential of the cell. Some studies have shown that folate restriction reduces 5-methyl-THF stores, alters DNA methyltransferase (DNMT) activity, and causes genomic hypomethylation. These effects have specific consequences for regulatory regions tied to metabolism, cell proliferation, and embryonic development [17,18].

While epigenetic writers determine the distribution of methyl marks, epigenetic readers such as MeCP2 determine how these marks are translated into transcriptional output. By binding methylated CpG-rich regions, MeCP2 recruits corepressor complexes and histone deacetylases, reinforcing chromatin compaction and limiting transcriptional plasticity. In metabolic contexts, the dysregulation of MeCP2 signaling may amplify transcriptional instability at genes involved in mitochondrial adaptation and neuroendocrine regulation of energy balance. This includes programs related to peroxisome proliferator-activated receptor gamma coactivator 1-alpha (PGC-1α) and brain-derived neurotrophic factor (BDNF)-dependent hypothalamic pathways. This reader layer is important, as aberrant DNA methylation may have limited phenotypic consequences unless it is effectively interpreted by chromatin-associated effector proteins [19].

In parallel, methionine—an essential amino acid and a direct precursor of SAM—plays a critical role in epigenetic control. The conversion of methionine into SAM allows the transfer of methyl groups to various biomolecules, producing SAH as a byproduct. The accumulation of this metabolite inhibits methyltransferase activity. The SAM/SAH ratio is a direct indicator of methylating capacity; moderate methionine restriction can decrease this ratio and lead to DNA hypomethylation in multiple tissues, whereas high methionine intake can induce the hypermethylation of sensitive genes. In both cases, one-carbon metabolism responds by epigenetically reprogramming genes associated with hepatic homeostasis, inflammation, and energy metabolism [7,14].

Likewise, vitamin B12 acts as an essential cofactor for methionine synthase, the enzyme responsible for the remethylation of homocysteine into methionine using 5-methyl-THF. Its deficiency impairs this reaction, reducing SAM availability and promoting SAH accumulation [15]. Recent evidence has revealed that even subclinical B12 deficiency may cause epigenetic alterations in metabolically active tissues; for example, low levels of vitamin B12 have been associated with the hypomethylation of genes related to mitochondrial function and energy homeostasis, as well as with epigenomic modifications, particularly during early developmental stages when deficiency occurs during pregnancy [20].

Meanwhile, choline and betaine act as alternative methyl donors in the betaine–homocysteine methyltransferase (BHMT)-catalyzed pathway, which is particularly relevant under conditions of low folate or low vitamin B12 availability. Choline, in addition to its structural role in membranes, can be oxidized into betaine to support homocysteine remethylation independently of the folate cycle. Choline availability during embryonic development exerts persistent epigenetic effects, including modifications in DNA methylation and changes in histone marks such as H3K9me2 and H3K27me3—epigenetic signatures typically associated with transcriptionally repressive chromatin states—as well as influencing neuronal programming and metabolism. Betaine additionally helps to restore a proper SAM/SAH balance and preserve DNMT activity, especially in the liver and kidneys [21].

Taken together, these metabolic pathways converge in the regulation of DNA methylation by DNA methyltransferases. DNMT1 is responsible for retaining methylation patterns during replication, while DNMT3A and DNMT3B generate de novo methylation in specific regions of the genome [22]. The activities of these enzymes depend on the availability of SAM and are inhibited by SAH, rendering them very sensitive metabolic sensors of nutrition and the state of one-carbon metabolism. It is known that nutritional variations can modulate DNMT expression in metabolic tissues, thereby altering epigenetic profiles associated with inflammation, adipogenesis, and mitochondrial dysfunction [23].

One of the most relevant metabolic effects of these nutritional/epigenetic interactions is regulation of the *PPARGC1A* gene, which encodes the transcriptional coactivator PGC-1α, a master regulator of mitochondrial biogenesis, oxidative metabolism, and glucose homeostasis. The methylation of its promoter is sensitive to fluctuations in folate, vitamin B12, methionine, and choline availability, and changes in *PPARGC1A* methylation are associated with insulin resistance, obesity, and metabolic dysfunction. Its expression can be altered by both hypermethylation and hypomethylation in specific promoter regions, significantly affecting the oxidative capacity of tissues such as skeletal muscle and liver [24].

Recent evidence supports the concept that one-carbon metabolism serves as a molecular bridge among nutrition, epigenetics, and energy metabolism. The availability of methyl-donating nutrients, the balance between SAM and SAH, and DNMT activity constitute a highly sensitive system that regulates the expression of genes that are key to cellular equilibrium. Alterations in any of these components can promote epigenetic reprogramming with long-term physiological consequences, particularly in metabolic tissues and during embryonic development, when epigenetic plasticity is at its highest [25].

These nutrients do not act as isolated inputs when viewed comparatively; rather, they support a shared methyl donor network with partially overlapping functions and tissue-specific relevance. Folate and vitamin B12 help determine methylation efficiency, methionine provides a direct substrate for SAM synthesis, and choline/betaine become important when folate-dependent flux is constrained. This integrated perspective explains how epigenetic impacts in obesity often reflect more than a deficiency of a single nutrient.

### 2.2. Histone Modifications and Nutrient Availability

Histone modifications play a key role in epigenetic regulation and are closely associated with nutrient availability and cellular energy levels. Modifications such as histone acetylation and deacetylation can modulate chromatin structure and gene transcription, enabling cells to respond to different stimuli. Nutritional metabolites, which include acetyl-CoA, NAD^+^, and SCFAs such as butyrate, function as essential cofactors or inhibitors of enzymes that write or erase histone marks through reversible post-translational modifications of histone tails [26].

Acetyl-CoA plays a central role in intermediary glucose and lipid metabolism. It donates acetyl groups for histone acetylation via HATs. Under nutrient-rich conditions, elevated intracellular acetyl-CoA levels promote an open chromatin state and activate genes involved in lipogenesis, mitochondrial biogenesis, and glucose metabolism. Conversely, fasting or caloric restriction can lower acetyl-CoA availability and favor deacetylation-mediated chromatin condensation [27].

SIRTs, particularly SIRT1, are NAD^+^-dependent histone deacetylases that function as metabolic sensors, orchestrating transcriptional responses to cellular energy status. SIRT1-mediated deacetylation is induced by caloric restriction, polyphenols, or elevated NAD^+^ levels. Under these conditions, it promotes mitochondrial biogenesis and oxidative metabolism by deacetylating transcriptional regulators, including PGC-1α and forkhead box O1 (*FOXO1*). In adipose tissue, SIRT1 suppresses adipogenesis by deacetylating peroxisome proliferator-activated receptor gamma (PPARγ) and promotes the browning of white adipocytes, thereby increasing both energy expenditure and insulin sensitivity [28,29].

Furthermore, nutrient-sensing pathways such as AMPK converge on SIRT1 to coordinate metabolic adaptations. When cellular energy is low, NAD^+^ accumulation triggers the AMPK phosphorylation response, which activates SIRT1. The AMPK–SIRT1 axis not only stimulates mitochondrial function, but also downregulates inflammatory genes via histone deacetylation and remodeling [30].

While the AMPK–SIRT1 axis is a key nutrient-sensing pathway, it should be considered within the wider context of chromatin regulation rather than as an independent epigenetic outcome. SIRT1 is better understood as an enzymatic mediator of nutrient-responsive deacetylation that links the cellular redox state to transcriptional remodeling, particularly in pathways involving mitochondrial function, adipocyte plasticity, and inflammatory restraint [30].

In addition to SIRT, HDACs exert repressive control over adipogenesis and metabolic gene programs; for example, HDAC9 expression is markedly increased in visceral adipose tissue during obesity and has been shown to inhibit adipocyte differentiation by interacting with transcription factors, including C/EBPα. It has been shown that HDAC9 deficiency enhances insulin sensitivity, reduces adipocyte hypertrophy, and protects against hepatic steatosis in some mouse models [31].

Moreover, butyrate—an SCFA produced by gut microbiota during dietary fiber fermentation—acts as a natural inhibitor of HDAC. Butyrate can induce hyperacetylation and activate genes involved in inflammation resolution, lipid metabolism, and epithelial barrier function by inhibiting histone deacetylases, thereby promoting histone acetylation. This fact provides strong evidence for the roles of fiber intake, microbiome-derived metabolites, and epigenetic regulation in metabolic tissues [32,33].

The microbiota-derived epigenetic signal is not restricted to butyrate. Propionate and acetate can influence chromatin states indirectly through acetyl-CoA metabolism and immune signaling, while less abundant metabolites such as valerate have emerged as candidate HDAC-modulating molecules with anti-inflammatory potential. As obesity-related metabolic dysfunction is sustained not only by excess lipid storage but also by chronic low-grade inflammation, gut-derived metabolites can shape epithelial integrity, immune tone, and histone acetylation landscapes [33].

Unlike acetylation, the functional impact of histone methylation depends on the residue and degree of methylation involved. Activating marks such as H3K4 methylation may participate in nutrient-sensitive transcriptional regulation, including pathways related to adipogenesis and hepatic metabolism. Enzymes such as SETD7 have been implicated in metabolic gene regulation, although their dietary responsiveness in obesity remains less well characterized than that of acetylation-related pathways. Evidence suggests that fluctuations in methyl donor availability, redox state, and intermediates may influence the activity of histone methyltransferases and demethylases in obesity [34,35].

Taken together, histone modifications constitute a metabolically sensitive epigenetic layer in which dietary factors modulate the activities of chromatin-modifying enzymes, thereby regulating gene expression in the context of obesity and related metabolic disorders. These mechanisms provide valuable targets for nutritional interventions directed at restoring metabolic homeostasis through epigenetic remodeling. Subsequent studies should further clarify how histone methyltransferases and demethylases may contribute to nutrient-responsive epigenomic remodeling.

### 2.3. MicroRNA and Nutrient Regulation

MicroRNAs (miRNAs) are small, non-coding RNAs that play a key role in post-transcriptional gene regulation by binding to complementary sequences on target mRNA, leading to mRNA degradation or translational repression. They act as key mediators of nutrient-sensitive gene expression, particularly in pathways regulating insulin signaling, adipogenesis, and lipid metabolism. Evidence suggests that dietary patterns and specific bioactive compounds can modulate miRNA expression profiles in metabolic tissues, thereby influencing susceptibility to obesity and related disorders [36,37].

Several diet-responsive miRNAs have been characterized within the context of metabolic regulation; for instance, miR-33a and miR-33b, when co-transcribed with the Sterol Regulatory Element-Binding Transcription Factor (*SREBF*) gene family, negatively regulate fatty acid oxidation and cholesterol efflux by targeting *ABCA1*, *CPT1A*, and *HADHB*. Overexpression of miR-33 suppresses lipid catabolism and promotes intracellular lipid accumulation, whereas its inhibition is associated with enhanced reverse cholesterol transport and improved insulin sensitivity [38,39].

On the other hand, miR-122 is a liver-enriched miRNA that plays a central role in hepatic lipid homeostasis. Its downregulation in obesity is associated with the increased expression of the genes involved in fatty acid oxidation, such as *CPT1*, thereby altering lipid flux and contributing to dyslipidemia. Experimental models have revealed that modulation of miR-122 levels may affect hepatic steatosis, insulin resistance, and systemic metabolic homeostasis [40,41,42].

Additionally, miR-223 is an inflammation-regulating miRNA that influences adipocyte differentiation and insulin responsiveness. It targets *FOXO1*, GLUT4, and *INSR*, serving as a buffer against inflammatory stress while maintaining adipocyte function under conditions of nutrient excess [43]. Dysregulation of miR-223 expression has been observed in both adipose and hepatic tissues of individuals with obesity and type 2 diabetes [44,45].

There is only preliminary evidence for transgenerational microRNA (miRNA) effects. Most studies are based on animal or developmental models, and direct human evidence is limited and often distorted by shared postnatal environmental exposures. For this reason, transgenerational microRNA signaling should be considered plausible, but not yet proven, in metabolic programming [46].

Apart from individual examples, global shifts in miRNA expression have been documented in response to dietary interventions, including caloric restriction, high-fat diets, and polyphenol supplementation. These compounds can indirectly modify miRNA transcription by altering chromatin accessibility or modulating upstream regulators, including sirtuins and histone-modifying enzymes [47]. However, the significance of the temporal dynamics governing when and how these dietary changes affect miRNA expression remains incompletely understood. Determining the optimal timing and duration of dietary modifications is crucial, as they may influence whether miRNA profiles shift in a more favorable direction or persist over time [48]. Without clarifying these temporal factors, it is challenging to ascertain the sustained impact of dietary interventions on miRNA-mediated metabolic regulation. Therefore, a systematic investigation of the timing of these miRNA responses is essential to identifying critical periods during which dietary interventions are most effective, ultimately informing the design and optimization of intervention protocols for precise and enduring health outcomes [47].

Taken together, these miRNA examples converge on three recurrent biological processes: attenuation of fatty acid oxidation, modulation of inflammatory signaling, and altered insulin sensitivity across liver and adipose tissue. This is important because single-miRNA findings are often context-dependent, while pathway-level convergence is more informative for biomarker development.

Therefore, diet-responsive miRNAs constitute a flexible post-transcriptional layer through which nutritional exposures can reshape the link between metabolic gene expression and diet. Their biomarker potential will depend on better standardization of sample source, temporal dynamics, and tissue concordance (Table 1).

Table 1 organizes nutrients, including folate, vitamin B12, choline, betaine, and methionine, as key methyl donors involved in one-carbon metabolism, thereby influencing DNA methylation capacity. It also details metabolic cofactors such as acetyl-CoA and NAD^+^, which are essential for histone acetylation and deacetylation. Furthermore, it outlines the modulatory effects of polyphenols, SCFAs, and other dietary compounds on epigenetic enzymes and non-coding RNA regulation. Collectively, these factors illustrate the multifaceted ways in which dietary factors modulate gene expression via epigenetic pathways.

These mechanistic layers provide the biological and biochemical basis for nutrient-sensitive chromatin regulation. However, their relevance becomes more evident when examined in metabolically active tissues, where epigenetic alterations are observed in obesity-relevant tissues, as discussed in the next section.

## 3. Epigenetic Regulators in Obesity and Adipose Tissue

### 3.1. Primary Epigenetic Enzymes

Epigenetic enzymes such as DNMT3A, TET2, SIRT1, and HDAC9 play central roles in adipose tissue metabolism and in the development and prevention of obesity-related dysfunctions. DNMT3A has been associated with adipose tissue insulin resistance due to its ability to methylate metabolically relevant loci, including the *FGF21* gene [54,55].

*FGF21* is relevant in this context because its expression is also epigenetically regulated. Experimental work has demonstrated that the *FGF21* locus undergoes PPARγ-dependent DNA demethylation in early life, with effects on inducible hepatic *FGF21* expression in adulthood. In obese humans, elevated circulating *FGF21* has been associated with leukocyte DNA methylation and microRNA signatures, supporting the view that it lies at the intersection of nutrient sensing, endocrine adaptation, and epigenetic memory [55].

In contrast, TET2 exerts demethylating activity and has been associated with improved insulin sensitivity and mitochondrial regulation. Interestingly, TET2 also regulates ß-adrenergic signaling by enhancing *Adrb3* transcription, a pathway essential for lipolysis and thermogenesis [55,56].

Additionally, SIRT1—a NAD^+^-dependent deacetylase—functions as a cellular energy sensor, promoting mitochondrial biogenesis and oxidative metabolism by deacetylating peroxisome proliferator-activated receptor gamma coactivator 1-alpha (PGC-1α). SIRT1 downregulates adipogenesis by deacetylating PPARγ, thereby decreasing white adipocyte hypertrophy and enhancing brown adipose tissue function. Its hepatic activity modulates lipid storage via AMPK/SREBP-1c signaling, and SIRT1 deficiency leads to steatosis, hyperglycemia, and oxidative stress [28].

HDAC9—a Class IIa histone deacetylase—acts as a negative regulator of adipocyte differentiation by forming repressive complexes that silence C/EBPα transcription. In obesity, its expression is upregulated in visceral adipose tissue, blocking adipogenesis and favoring hypertrophic expansion over hyperplasia. Deletion of HDAC9 in mice resulted in smaller, functional adipocytes, increased thermogenic gene expression (e.g., *UCP1*), and improved insulin sensitivity. Moreover, hepatic *HDAC9* depletion enhanced β-oxidation and reduced lipid accumulation, suggesting that *HDAC9* suppression confers tissue-specific benefits [31].

Across adipose and hepatic tissues, a common regulatory mechanism involving epigenetic dysfunction tends to suppress oxidative and thermogenic programs while favoring hypertrophy, ectopic lipid storage, and insulin resistance, collectively known as obesity. DNMT3A and HDAC9 reinforce the transcriptional repression of metabolic favorable states, where TET2 and SIRT1 counterbalance these effects by supporting mitochondrial competence and adaptive remodeling. Thus, these enzymes clarify the importance of tissue context in biomarker interpretation and therapeutic targeting.

### 3.2. Epigenetic Signatures for Methylation

Epigenetic signatures in adipose and hepatic tissues have emerged as a key regulatory factor in metabolic disorders related to obesity. Consistent links have been found between specific DNA methylation patterns in genes such as PPARγ, leptin (*LEP*), adiponectin (*ADIPOQ*), and insulin receptor substrate 1 (*IRS1*), which have been associated with alterations in insulin sensitivity, adipocyte function, and hepatic lipid metabolism [3].

Tissue-specific findings should be distinguished from blood-based methylation signals and other metabolically relevant loci, such as *PPARGC1A*, which are more closely linked to mitochondrial regulation than to adipocyte differentiation [24].

In human visceral and subcutaneous adipose tissue, PPARγ promoter hypermethylation has been associated with reduced gene expression and impaired adipogenesis. This epigenetic silencing precedes other metabolic dysfunctions, including adipocyte insulin resistance and lipid accumulation. Furthermore, in hepatic tissue, saturated fat-rich diets have been shown to induce PPARγ promoter hypomethylation, thereby enhancing transcription of fatty acid transporter genes such as *CD36*, hence supporting de novo lipogenesis and hepatic steatosis [57]. Interestingly, hypermethylation of PPARγ in peripheral blood has also been proposed as a biomarker for the severity of fibrosis in non-alcoholic fatty liver disease (NAFLD) [58].

In turn, the *LEP* gene exhibits an inverse methylation pattern. In obesity, *LEP* promoter hypomethylation correlates with increased gene expression, leading to the hyperleptinemia frequently observed in insulin-resistant individuals. Studies in both adipose tissue and leukocytes have demonstrated that lower *LEP* methylation levels are associated with increased BMI, waist circumference (WC), and Homeostatic Model Assessment of Insulin Resistance (HOMA-IR) scores. Weight loss and dietary interventions appear to partially reverse these methylation patterns, restoring leptin expression control [59].

Similarly, *ADIPOQ*, which encodes the adipokine adiponectin, shows hypermethylation in obese and diabetic subjects, particularly in the CpG-enriched promoter regions of subcutaneous fat. This epigenetic repression is associated with decreased adiponectin levels, impaired glucose uptake, and insulin resistance. The epigenetic silencing of *ADIPOQ* also abolishes its protective effects in the liver, promoting the uptake of free fatty acids and hepatic lipid deposition in NAFLD [60].

Methylation changes in *IRS1*—a key insulin signaling adaptor—also play an essential role, with increased promoter methylation of *IRS1* having been reported in the visceral adipose tissue of individuals with obesity; in particular, this was connected with reduced mRNA expression and higher waist-to-hip ratios. These alterations disrupt downstream insulin signaling and serve as epigenetic indicators of peripheral insulin resistance [57,58,59,60,61].

Collectively, these DNA methylation patterns (either hyper- or hypomethylation) highlight the tissue-specific epigenetic dysregulation associated with obesity and metabolic syndrome [62]. These signatures have potential utility as predictive and diagnostic biomarkers, particularly when integrated into multi-omics datasets to enable more refined metabolic profiling.

Although these locus-specific findings are biologically relevant, several limitations should be considered. Some limitations include the fact that studies are cross-sectional, sample sizes are modest, and methylation measurements are often derived from blood or mixed adipose samples rather than from purified cell populations. Others, such as medication use, smoking status, inflammatory burden, and effects of cell composition, may also influence the observed associations. Therefore, epigenetic marks are better viewed as candidate biomarker signals than causal mediators [61].

Markers with the most consistent metabolic correlations across the literature are summarized in Table 2. Consistency should not be confused with clinical readiness, as few of these candidates have undergone prospective validation or a formal assessment of their predictive value relative to conventional risk markers.

Importantly, reproducibility across studies should also not be interpreted as clinical readiness, as most candidate loci lack prospective validation and standardized implementation frameworks.

## 4. Nutrient/Epigenetic Interactions: Evidence from Human Studies

### 4.1. Dietary Patterns and Epigenomic Profiles

In recent decades, the diet has been recognized as a key determinant of health, influencing metabolism, immune function, cognitive performance, and disease risk. When consumed, food is absorbed and metabolized into the bloodstream as bioactive components, playing different roles in the prevention or development of diseases [12]. While the main focus has been on early or medium-term outcomes, the diet can also exert long-term effects through multiple mechanisms, with epigenetic modifications being among the most important. Although it may appear simple to evaluate, the diet is complex and constantly changing, shaped by biological, cultural, and environmental factors in humans [48]. This renders it a complex object of study that can be classified as a qualitative variable, such as the “Mediterranean” or the “DASH” diet, or as a quantitative variable, with the focus on estimating nutrient intake or the type of food processing [64].

An “obesogenic diet” refers to a dietary pattern characterized by sustained positive energy balance and high intake of ultra-processed foods, refined carbohydrates, and saturated fats, leading to epigenetic changes at loci involved in appetite regulation, adipogenesis, and immune signaling. However, the available evidence in humans remains heterogeneous, and many reported methylation changes may reflect existing metabolic dysfunction rather than primary dietary effects [65].

By contrast, the Mediterranean diet has been associated with healthier epigenetic profiles, driven by changes in DNA methylation and chromatin modifications that regulate genes linked to inflammation, metabolism, and aging. This may partly explain the beneficial epigenetic effects in preventing cardiometabolic and chronic diseases [66]. Among the main components of this diet are olive oil, which is rich in polyphenols and monounsaturated fatty acids, and fish, which contain polyunsaturated fatty acids such as omega-3 fatty acids, which have been associated with the methylation of genes related to inflammatory effects and metabolic functions [67]. Another effect of this diet is a reduction in the risk of chronic diseases, attributable to the hypermethylation of proinflammatory genes and the regulation of protective genes. The effect of this diet on histone modification has also been highlighted, which is mediated through several bioactive components—including resveratrol in grapes and wine; catechins in fruits, wine, cocoa, and green tea; and the fatty acids in dressings and fish—which may stimulate the activation of enzymes such as HAT and HDAC, thus promoting the expression of antioxidant and anti-inflammatory genes [67]. Some clinical studies have indicated that the Mediterranean diet reduces the expression of proinflammatory genes through epigenetic changes, thereby reducing the risk of metabolic disorders [50].

Tomé-Carneiro et al. examined a sample of subjects with type 2 diabetes and hypertension who participated in a 1-year study comparing the consumption of grape extract enriched with 8 mg of resveratrol with the consumption of grape extract or maltodextrins [50]. Changes in serum alkaline phosphatase concentrations of 16.1% and 13.4% were observed in the grape extract and enriched extract groups, respectively, with a 13.3% reduction in IL-6 observed in the latter group. The authors also noted a decrease in proinflammatory cytokines, including CCL3, IL-1β, and TNF-α. Therefore, long-term resveratrol supplementation suppresses the expression of proinflammatory cytokines associated with circulating immune cells in hypertensive patients with type 2 diabetes.

Hoffmann et al. conducted a randomized controlled trial (RCT) evaluating the effect of a polyphenol-enriched Mediterranean diet on methylome and transcriptome, with the intent of assessing the molecular mechanisms underlying metabolic improvements after 18 months [66]. The authors found that subjects who received a polyphenol-enriched Mediterranean diet low in red/processed meat showed 6% more epigenetically modified transcriptional modulator genes than those on other diets, and these changes correlated positively with changes in the subcutaneous fat area, body weight, and WC [68].

On the other hand, Tehrani et al. studied the effect of a Mediterranean diet supplemented with olive oil and fresh fruit in adults and older adults with high cardiovascular risk. After a 3-month intervention, these researchers observed that the Mediterranean diet with olive oil decreased the expression of cyclooxygenase-2, low-density lipoprotein receptor-related protein 1, and monocyte chemotactic protein 1 [69]. Meanwhile, the Mediterranean diet with fresh fruit increased the expression of *CD36* and the tissue-factor pathway inhibitor. Therefore, this type of diet may help to modulate the expression of proatherothrombotic genes and those related to vascular inflammation [70].

Additionally, the DASH diet has been associated with favorable epigenetic profiles, as reflected in DNA methylation, along with the histone modifications of genes involved in methylation, blood pressure, and lipid metabolism. This is achieved by emphasizing fruits, vegetables, and whole grains as primary dietary sources, thus promoting the hypermethylation of proinflammatory genes and improving the regulation of antioxidant genes. It has also been suggested that this type of diet can influence the epigenetic processes of aging and slow the body’s degenerative processes [71]. Similar to the Mediterranean diet, it is rich in polyphenols and minerals that modulate HAT activity, thereby activating cardioprotective genes. This occurs through identified changes in the regulation of endothelial function and the renin–angiotensin–aldosterone system [13].

Collectively, cardioprotective dietary patterns, such as the Mediterranean and DASH diets, share several epigenetically relevant features, including higher polyphenol intake, greater fiber intake, and improved micronutrient quality. Across studies, these patterns are more consistently associated with reduced inflammatory signaling than with any single, reproducible methylation change. The strongest current evidence supports pathway-level modulation rather than universal diet-specific epigenetic signatures.

In contrast, Western dietary patterns accelerate epigenetic aging and activate proinflammatory pathways [72]. Furthermore, decreased folate and vitamin B12 intake proportionally reduces the number of methyl group donors, altering methylation and immune response regulators. Among the various consequences of the latter is the persistence of unfavorable epigenetic patterns associated with increased cardiometabolic and chronic disease risk [73].

On the other hand, a vegetarian diet confers benefits such as reduced signs of biological aging and the favorable regulation of anti-inflammatory genes [68]. Clinical trials involving vegetarian diets in a sample of identical twins revealed that this type of diet reduced biological age, as estimated with DNA methylation. Similarly, some population-based studies have indicated that vegetarian diets are associated with a lower epigenetic age. This may be due to the higher intake of polyphenols and antioxidants that modulate anti-inflammatory genes and pathways linked to longevity, such as *FOXO3* and SIRT1, which are involved in metabolism and stress resistance, thereby reducing cardiovascular and metabolic risk through epigenetic modifications [72].

Emerging data also highlight the influence of biological sex on nutrient/epigenetic interactions, with sex-specific DNA methylation patterns reported in response to dietary interventions. These observations strengthen the importance of including sex-stratified analyses in future epigenomic studies in order to better understand the heterogeneity of metabolic disease. For instance, women may benefit from customized folate guidelines to optimize their epigenetic profiles, potentially improving outcomes under specific metabolic conditions. Implementing sex-specific dietary recommendations can translate theoretical observations into practical solutions that enhance personalized nutrition strategies [74].

These dietary effects may, in part, be mediated through gut microbial metabolites such as butyrate and propionate, which are known to influence host histone acetylation and DNA methylation patterns [75]. Given the heterogeneity of dietary exposures, Table 3 synthesizes dominant dietary patterns and their associated epigenetic and metabolic signatures.

Even so, the intervention literature remains limited by small sample sizes, heterogeneous endpoints, incomplete control for weight loss as a confounder, and limited multi-tissue sampling. Accordingly, these studies support biological plausibility but do not yet define clinically deployable epigenetic biomarkers.

### 4.2. Dietary Intervention Trials and Epigenetic Regulation

Human studies have suggested that folate-related interventions may modify DNA methylation profiles, as one-carbon metabolism determines the availability of methyl groups. This concept is supported by population-level evidence on folate status and randomized intervention studies evaluating folic acid supplementation; however, the clinical significance of these methylation changes remains uncertain [77,78,79].

In addition, both docosahexaenoic acid (DHA) and eicosapentaenoic acid (EPA) can modulate histone acetylation by regulating HAT and HDAC, hence altering the expression of genes associated with inflammation, metabolism, and cancer [80]. These effects manifest through changes in chromatin structure, where modulation of HAT and HDAC by DHA and EPA occurs, consequently altering gene expression. More specifically, DHA increases acetylation at residues such as H3K9Ac, H4K5Ac, H4K8Ac, H4K12Ac, and H4K16Ac, thereby promoting an open chromatin state and the increased expression of protective genes [80].

Intervention studies have suggested that methyl donor availability and polyphenol-rich exposures can modulate the epigenetic machinery through distinct but complementary routes: folate primarily affects methyl group supply and DNA methylation capacity, whereas polyphenols and omega-3 fatty acids more often influence histone acetylation, deacetylase activity, and inflammatory transcriptional programs [80].

Other substances, such as resveratrol, curcumin, and epigallocatechin gallate (EGCG), are polyphenols that can act as epigenetic modulators, regulating the activity of HDAC and SIRT. These polyphenols can increase histone acetylation, activate tumor suppressor genes, and enhance antioxidant and anti-inflammatory responses [52]. Resveratrol can specifically activate SIRT1, an NAD-dependent deacetylase that exerts an influence on longevity, metabolism, and the stress response. It also affects HDAC, promoting histone acetylation and the expression of protective genes. Curcumin can inhibit certain HDACs, promoting histone acetylation and the expression of protective genes, and can modulate SIRT1 and other SIRTs, hence improving epigenetic regulation of inflammatory and metabolic functions [53].

Taken together, human studies support the biological plausibility of the abovementioned mechanisms, while also exposing the limits of single-layer interpretation, motivating the need for multi-omics integration and predictive modeling.

## 5. Combining Multi-Omics Approaches to Validate Predictive Epigenetic Signatures

### 5.1. Epigenome-Wide Association Studies in Obesity

EWAS have charted the relationship between DNA methylation (DNAm) and distinctive adiposity-related traits, providing a molecular interpretation with promising clinical utility. In adults, a multi-cohort meta-analysis (~10,261 participants) reported 187 CpG sites associated with BMI. The results of directionality analyses and Mendelian randomization (MR) have indicated that adiposity generally precedes many epigenetic changes. Some signals, including methylation sites linked to gene expression through quantitative trait loci (eQTLs), are associated with gene expression and a methylation risk score (MRS)-predicted type 2 diabetes, over standard clinical covariates [51].

A subsequent transethnic meta-analysis (*n* ≈ 17,034; external replication ≈ 4822) expanded the catalogue to over >1000 CpGs and derived an epigenetic BMI (eBMI) that captures cardiometabolic heterogeneity (fasting glucose, triglycerides, and HDL cholesterol) that is not fully reflected by BMI, explaining approximately 32% of the variation in BMI in the test sample [11].

In pediatric cohorts (23 studies; *n* ≈ 4133), associations are smaller but show increasing convergence with adult patterns with advancing age. These results suggest that blood DNAm acts as an integrator of accumulated adiposity exposure, rather than a primary driver [63]. For waist circumference (WC), discovery/replication analyses in the Rotterdam Study (RS) with validation in the Atherosclerosis Risk in Communities (ARIC) identified signals and added loci with cross-ancestry replication. Overall, these datasets support the use of blood-based epigenetic signatures as systemic biomarkers of metabolic status. In practice, this requires confirming tissue specificity (blood vs. adipose), controlling for key confounders (smoking, medications, leukocyte composition), and integrating transcriptomics and metabolomics to identify markers with biological function and actual clinical utility [63].

In the case of BMI, consistent signals converge on *ABCG1* (*cg06500161*), *CPT1A* (*cg00574958*), and *SREBF1* (*cg11024682*), mechanistic nodes that link the clinical phenotype to cholesterol efflux, fatty acid β-oxidation, and lipogenesis, respectively [81]. These blood-based associations, supported by eQTM connections, also relate to lipid profiles and inflammatory markers, refining cardiometabolic risk beyond anthropometry [63].

For WC, RS/ARIC analyses report associations at *LGALS3BP*, *MAP2K3*, *DHCR24*, *CPSF4L*, and *TMEM49*, and identify shared loci between BMI and WC (*MSI2*, *LARS2*), pointing to the roles of immunometabolism and membrane-remodeling pathways in central adiposity [11]. In the context of clinical nutrition, sustained weight loss interventions can produce modest, site-specific changes in the DNAm of genes that help to manage lipid metabolism and inflammation. While these effects are partial and tissue-dependent, they often coincide with improvements in the cardiometabolic profile and insulin sensitivity, positioning these loci as candidates for future biomarker panels that require functional validation, longitudinal testing, and demonstration of incremental predictive value [82].

Despite their reproducibility, EWAS signals should be interpreted with caution. While blood-derived methylation profiles can capture systemic exposure, they do not fully resolve tissue-specific biology in adipose tissue, liver, or muscle. Persistent concerns include smoking, age, inflammation, medication use, and leukocyte composition, and reverse causality is relevant because adipose tissue itself can reshape the methylome. To enable clinical translation, prospective validation and incremental prediction analyses beyond standard risk factors will be required in future studies. Harmonized preprocessing lines will also be necessary.

### 5.2. Machine Learning and Systems Biology Methods

Multi-omics integration has emerged as an indispensable framework for understanding the complexity of biological systems, particularly for linking gene regulation, metabolic state, and functional cellular phenotypes. Among the possible combinations, the integration of transcriptomics, metabolomics, and methylomics occupies a central place, as it directly links three complementary levels of regulation: gene expression, epigenetic control of that expression, and the resulting metabolic activity [83].

Although transcriptomics, methylomics, and metabolomics can be examined independently, their integration provides a systems-level perspective linking epigenetic regulation, gene expression, and metabolic activity [84]. Metabolomics, by contrast, represents the final functional manifestation of these regulatory processes, capturing changes in biochemical pathways and cellular energy states. The coordinated merging of these three modalities enables the correlation of DNA methylation patterns with changes in gene expression and specific metabolic alterations, affording a functionally integrated view of cellular and tissue status [83,84].

The development and application of this type of integration have been enabled by the advent of large-scale data analysis, understood not only as the ability to generate large volumes of data but also as the set of data-processing methods required to process, harmonize, and interpret them jointly. High-throughput omics technologies, including single-cell platforms and spatial multi-omics approaches, have generated high-dimensional, heterogeneous datasets that obtain transcriptomic, epigenomic, and metabolomic information at exceptional resolution [85].

In this context, high-throughput screening becomes a structural component of multi-omics integration, enabling alignment across molecular modalities with different scales and distributions. Several studies have indicated that the effective integration of transcriptomics, methylomics, and metabolomics requires high-level computational methods, such as dimensionality reduction, multivariate statistical models, and machine learning approaches capable of identifying nonlinear relationships across molecular layers. These strategies facilitate the creation of hidden representations of the cellular state that preserve biologically relevant information and minimize technical biases [86].

Specifically, in single-cell and spatial multi-omics scenarios, high-throughput assessment permits the connection of cellular heterogeneity, epigenetic regulation, and metabolic activity within a defined tissue context, thereby improving the identification and validation of predictive molecular signals [85,87].

Computational integration is most informative when it links an epigenetic signal to a downstream biological phenotype; for example, clustering CpG methylation at lipid-related loci with transcriptomic and metabolomic features can help to determine whether a methylation signature reflects fatty acid oxidation, inflammatory tone, or adipokine dysregulation. Likewise, models that derive methylation risk scores or epigenetic BMI estimates become biologically meaningful when their key features can be traced back to pathways such as cholesterol transport, mitochondrial function, or hepatic lipid handling [85].

However, challenges arise related to technical heterogeneity across platforms, handling missing data, and the considerable computational cost of large-scale analyses [86]. Despite these limitations, the convergence of high-resolution multi-omics technologies and large-scale analyses continues to expand the capacity of these approaches, establishing them as essential tools for translational research and precise, accurate medicine [10].

For machine learning outputs to move beyond statistical classification toward mechanistically credible biomarkers, model interpretability and external validation may provide an alternative for bridging translational gaps.

## 6. Translational Implications: Bridging Molecular Insights and Clinical Practice

This section transitions the discussion from mechanistic and experimental findings to their applications in clinical and public health contexts. It explores how advances in nutrient-sensitive epigenetics can inform individualized nutrition, precision medicine, and early-life prevention strategies. By synthesizing evidence across molecular, multi-omics, and population-based studies, the following sections address (1) the use of epigenetic profiles to guide precision nutrition at the individual level, and (2) how understanding epigenetic mechanisms can outline stronger public health approaches and early-life programming. These translational perspectives underscore the challenges of applying molecular knowledge to real-world interventions, from clinical trials to public health policy.

### 6.1. Precision Nutrition According to Epigenetic Profiles

Precision nutrition aims to customize dietary strategies for individual biological profiles, integrating genetic-, epigenetic-, metabolic-, and microbiome-derived data to improve health outcomes and prevent chronic disease. Within this system, molecular markers are considered dynamic tools for stratifying patients according to their metabolic risk and responsiveness to diet-based interventions [88]. However, existing epigenetic biomarkers remain primarily research tools, as most lack prospective validation and standardized assays, and their predictive value beyond conventional clinical markers has not yet been demonstrated.

From a public health perspective, the value of epigenetic profiling lies in targeted prevention research and risk refinement in high-risk groups, rather than in universal screening. Assays intended for broader use must demonstrate analytical robustness, affordability, cross-population reproducibility, and transparent governance regarding consent, data use, and unbiased access. Until these standards are met, epigenetic profiling should be viewed only as a research tool, rather than an established clinical screening platform [89,90,91].

Thus, advances in single-cell epigenomics and high-definition spatial profiling may allow for the characterization of intratissue heterogeneity in response to nutrients. These data could enable the development of fine-tuned nutritional interventions that account for tissue-specific epigenetic states, with possible applications in metabolic surgery follow-up, weight loss maintenance, and early risk prediction in pre-diabetic or child populations [92].

Any high-level nutritional care strategy guided by epigenetic data must be developed alongside considerations of equity, bioethics, and psychological readiness. As the field advances toward clinical utility, it will be essential to translate molecular scores into accessible, culturally appropriate tools to avoid exacerbating health disparities [93]. Educational efforts, ethical regulation, and oversight of direct-to-consumer epigenetic services are necessary. These emerging technologies are applied safely, effectively, and fairly [94].

### 6.2. Public Health and Early-Life Programming

Beyond individual-level applications, epigenetic insights also have transformative potential in public health, particularly for early-life nutritional programming [95]. The concept of the Developmental Origins of Health and Disease (DOHaD) suggests that maternal nutrition, stress, and environmental exposures during pregnancy and early childhood can induce long-lasting epigenetic changes that impact disease susceptibility throughout life [96].

Maternal intake of methyl donors (such as folate, vitamin B12, and choline), polyunsaturated fatty acids (PUFAs), and polyphenols has been shown to influence epigenetic methylation and histone modification patterns in fetal tissues, with effects on adipocyte differentiation, insulin signaling, and organogenesis [97]. These early epigenetic imprints contribute to intergenerational transmission of metabolic risk and, conversely, represent a powerful window for preventive interventions [98,99].

Programs providing prenatal supplements or targeted dietary interventions during lactation may epigenetically prime offspring to have better cardiometabolic outcomes. This has important implications for public health policy, highlighting critical periods of susceptibility and establishing evidence-based guidelines for maternal and infant nutrition [100].

Early-life epigenetic programming may also involve neuroendocrine pathways that regulate appetite, satiety, and inflammation. Maternal diet and metabolic status can influence the epigenetic regulation of hypothalamic circuits by modulating the energy balance and shaping lifelong vulnerability to hyperphagia, adiposity, and insulin resistance. Together, these factors provide a direct translational structure for the broader socio-behavioral considerations [100].

Finally, epigenetics provides a mechanistic link between nutrition and long-term health trajectories. Its incorporation into precision nutrition and public health programs holds considerable potential for research but warrants careful examination of the ethical, developmental, and sociocultural dimensions of any given country [101].

## 7. Future Directions and Research Gaps

A major conceptual gap in nutritional epigenetics is the inadequate distinction between systemic epigenetic signatures determined in accessible tissues and tissue-specific epigenetic remodeling occurring in metabolically active organs, such as adipose tissue, liver, skeletal muscle, and the intestinal epithelium [54,95,99]. Future work should determine whether blood-based signatures can serve as markers of systemic exposure or direct measures of epithelial, hepatic, adipose, or hypothalamic adaptation [75].

A more explicitly mechanistic approach is now required for the microbiome/epigenome axis. Future studies should move beyond descriptive accounts of SCFAs and test specific hypotheses regarding their distribution along the gut axis, bile acid receptor signaling, and the role of low-abundance microbial metabolites in shaping epithelial chromatin states, mucosal immunity, and systemic inflammation.

A second priority concerns temporal stability. While changes in diet may be transient, reflecting adaptive responses, others may persist as epigenetic molecular memory [91]. This has direct clinical relevance, as reversible marks define intervention possibilities, while persistent marks may be useful for risk stratification or identifying individuals with prolonged metabolic imprinting [102].

Therefore, longitudinal human studies—especially those beginning in early life and extending through dietary transitions—are critical for evaluating the stability and reversibility of epigenetic signatures. Integrating omics data across time points will be essential for tracking the biological memory encoded by diet and its downstream functional effects [103].

Notwithstanding, causal validation remains another obstacle. CRISPR-based epigenome editing, locus-specific reporter systems, and lineage-tracing approaches provide tools to test whether methylation or histone marks may drive metabolic phenotypes [104]. However, these methods have several limitations, including off-target effects, incomplete tissue specificity, delivery constraints, and ethical barriers to human application [49]. Therefore, their value will depend on careful use of focused preclinical systems.

Model development should certainly be prioritized. Intestinal, adipose, and hepatic organoid systems, as well as cell or spatial multi-omics platforms, can be used to map the relationships between nutrient doses and cell-type-specific epigenetic responses under certain physiological conditions. To this end, these model systems should be integrated with clinical cohorts to improve translational relevance [94,105,106,107,108].

Finally, the field may consider adopting epigenetic biomarkers, which will require more than biological plausibility. Standardized preprocessing pipelines, governance frameworks, and bioethical standards should be developed in parallel to ensure these tools are applied in ways that are both scientifically valid and socially responsible. Questions of data ownership, equitable access, and responsible communication should therefore be addressed in parallel with biomarker development [54,109,110].

## 8. Discussion

This review integrates mechanistic, human, and multi-omics evidence to establish nutrient-sensitive epigenetic regulation as a biomarker interface rather than as a set of individual molecular observations. Although the available evidence demonstrates biological plausibility, clinical implementation is not yet supported. Even the strongest human evidence supports pathway-level convergence and candidate biomarker panels, rather than single-marker clinical prediction. Meaningful translation will depend on tissue-aware validation, longitudinal studies, and a tailored assessment of public health feasibility [84].

Importantly, this pathway-level convergence is supported by a restricted set of recurrent methylation loci identified across EWAS, which includes *ABCG1*, *CPT1A*, and *SREBF1*. These loci provide molecular anchors linking epigenetic variation to key metabolic processes such as cholesterol transport, fatty acid β-oxidation, and lipogenesis [81].

One of the most important insights is the molecular convergence of nutrient-derived metabolites with chromatin-modifying enzymes. One-carbon metabolism emerges not simply as a biochemical pathway but as a nutrient-responsive epigenetic sensor that modulates methyl donor availability and the SAM/SAH ratio, with effects on DNA and histone methylation in metabolically active tissues [17,19]. These methylation signatures are not isolated epiphenomena. Rather, they reflect the transcriptional memory of nutritional states, shaping adipocyte identity, mitochondrial output, and insulin responsiveness [8,24]; however, these modifications remain a matter of debate, particularly in human studies, where distinguishing cause from effect is particularly difficult.

Similarly, the roles of histone acetylation and deacetylation demonstrate how intracellular energy status and NAD^+^ availability govern chromatin accessibility and metabolic gene expression [6,91]. SIRT1-mediated deacetylation links nutrient sensing to mitochondrial biogenesis and inflammatory restraint [13]. These effects are highly tissue- and context-dependent and are frequently influenced by age, sex, and the hormonal milieu—variables which are often overlooked in standard designs [27,54].

From a translational standpoint, the biomarker value of miRNA depends on the compartment. Circulating miRNAs are attractive because they are accessible and potentially scalable, but they also integrate signals from multiple tissues and inflammatory cell populations. Tissue-specific miRNA measurements may offer mechanistic precision for liver and adipose tissue, but are less feasible for routine clinical use. Resolving the inconsistency between circulating and tissue miRNA signals will be essential before these markers can be incorporated into biomarker panels [110].

Although EWAS data provide reproducible associations, the interpretation depends on methods that can address causality. Longitudinal EWAS, Mendelian randomization, and intervention-responsive methylation analyses may also clarify which signals track cumulative adiposity, which precede metabolic deterioration, and which are most susceptible to confounding factors. Nevertheless, only a few loci have been functionally validated and there is still limited prospective evidence for clinical deployment.

Studies investigating the effects of the Mediterranean and DASH diet remain consistent, showing beneficial epigenetic effects on inflammatory and metabolic phenotypes alongside plausible molecular shifts. However, most trials have limited duration, lack multi-tissue sampling, include insufficient controls, and often fail to improve clinical markers, making it too early to define diet-specific epigenetic signatures for clinical use. Emerging epigenetic clocks offer systemic but imperfect substitutes, although their concordance with metabolic tissue remodeling remains controversial.

Moreover, while blood is widely used for screening because it is accessible, it cannot be used as a substitute for metabolic tissues. Moving forward, a combination of minimally invasive approaches (e.g., blood-based markers) and computational innovation based on high-quality reference methylomes is required. The aim should be to develop clinically interpretable substitute markers, rather than reconstructing tissue.

Perhaps most critically, the field must acknowledge its technological and ethical limits. The appeal of epigenetic profiling for risk prediction and personalized nutrition is unquestionable, but its translation into practice requires rigorous validation, cost-effectiveness analyses, and explicit communication with patients. At the same time, public discourse must avoid deterministic framings that reduce complex social, dietary, and biological interactions to overly simplistic narratives of gene regulation.

This study reinforces the notion that nutrient-sensitive epigenetic regulation is not only a molecular bridge between diet and metabolic health, but also a bidirectional, context-adaptive interface capable of encoding past exposures and regulating future responses. The clinical value of this paradigm depends not only on molecular resolution but also on the effectiveness with which dynamic, individualized, and ethically sound frameworks for prevention and care are integrated.

## 9. Conclusions

Nutrient-sensitive epigenetic regulation is not merely an additional layer of metabolic control. Rather, it is a mechanistic interface through which dietary exposures may influence mitochondrial function, adipose remodeling, inflammatory signaling, and insulin sensitivity. The most compelling processes that impact key transcriptional programs arise from convergent patterns rather than from any single marker, particularly recurrent blood-based methylation loci such as *ABCG1*, *CPT1A*, and *SREBF1*, adipokine-related regulatory regions, and selected diet-responsive microRNA connections.

Clinical implementation remains premature. Major barriers include tissue specificity, assay standardization, inter-cohort reproducibility, cost, and the limited availability of prospective studies with transcriptomic and metabolomic data. In the near-term, the most feasible translational application is the development of validated biomarker panels for research stratification and early risk refinement.

Understanding nutrient-sensitive epigenetic signatures offers a basis for linking diet patterns to metabolic phenotype with greater mechanistic depth, instead of relying on clinical measures alone. Future clinical relevance will depend on the ability to move from descriptive associations to reproducible, tissue-specific, and clinically validated evidence.

**Figure 1:**
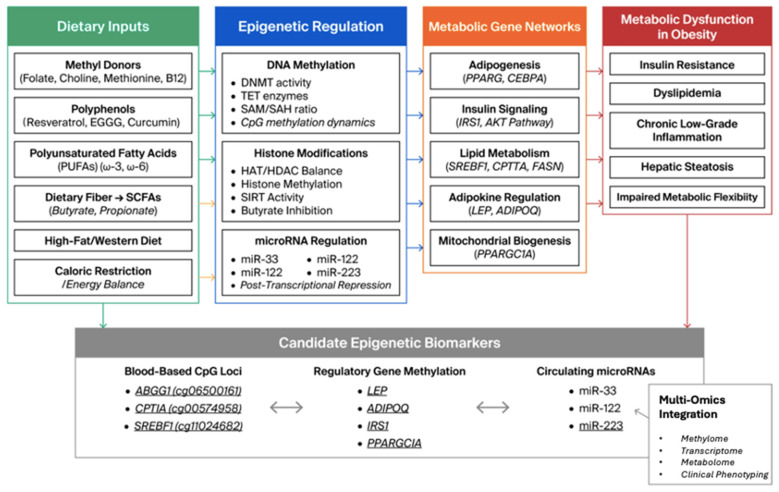
Integrative framework of nutrient-sensitive epigenetic regulation in obesity.

As shown in Figure 1, the proposed framework links dietary inputs to epigenetic regulation, downstream metabolic pathways, and the biomarker layers most likely to support translational development.

## Figures and Tables

**Table 1:** Major nutrient classes and their epigenetic mechanisms in metabolic regulation.

Nutrient/Compound	Epigenetic Mechanism	Epigenetic Effect	References
Folate, vitamin B12, methionine, choline, betaine	One-carbon metabolism, regulation of SAM/SAH ratio; DNMT activity	Modulation of DNA methylation capacity and genome-wide methylation patterns	[49]
Acetyl-CoA	Substrate for histone acetyltransferases (HATs)	Increased histone acetylation and transcriptional activation of metabolic genes	[26,27]
NAD+ (e.g., fasting, polyphenols)	Cofactor for sirtuins (SIRT1)	Histone deacetylation and regulation of mitochondrial and metabolic gene expression	[28,30]
Short-chain fatty acids (butyrate, propionate)	HDAC inhibition, indirect effects on acetyl-CoA metabolism	Increased histone acetylation; modulation of inflammatory and metabolic pathways	[32,33]
Omega-3 fatty acids (DHA, EPA)	Modulation of HAT/HDAC activity; chromatin remodeling	Regulation of inflammatory and lipid metabolism genes via histone acetylation changes	[49,50,51]
Polyphenols (resveratrol, curcumin, EGCG)	Modulation of SIRT1 and HDAC activity; chromatin remodeling	Regulation of antioxidant, anti-inflammatory, and metabolic gene expression	[52,53]
Diet-responsive miRNAs	Post-transcriptional gene regulation (mRNA targeting)	Modulation of lipid metabolism, inflammation, and insulin signaling pathways	[36,37]

**Table 2:** Representative epigenetic modifications associated with obesity-related metabolic dysfunction.

Epigenetic Modification	Molecular Effect	Metabolic Consequence	References
*PPARGC1A* promoter hypermethylation	Reduced PGC-1α expression	Impaired mitochondrial biogenesis and oxidative metabolism, insulin resistance	[21]
SIRT1-mediated deacetylation	Activation of PGC-1α and *FOXO1* pathways	Increased mitochondrial function, improved insulin sensitivity, anti-inflammatory effects	[28,30]
miR-33 overexpression	Inhibition of *ABCA1*, *CPT1A*, *HADHB*	Reduced fatty acid oxidation and cholesterol efflux, increased lipid accumulation	[38,39]
*LEP* promoter hypomethylation	Increased leptin expression	Hyperleptinemia, leptin resistance, association with obesity and insulin resistance	[8,59]
*ABCG1* methylation (blood EWAS)	Altered cholesterol transport gene regulation	Associated with BMI, dyslipidemia, cardiometabolic risk	[51,62,63]
*CPT1A* methylation (blood EWAS)	Altered fatty acid β-oxidation	Associated with adiposity and metabolic dysfunction	[51,62,63]
*SREBF1* methylation (blood EWAS)	Dysregulation of lipogenesis pathways	Associated with lipid metabolism alterations and obesity risk	[51,62,63]

**Table 3:** Dietary patterns, associated epigenetic mechanisms, and reported metabolic outcomes.

Dietary Pattern	Epigenetic Mechanism	Reported Metabolic Effects	References
Mediterranean diet	Modulation of inflammatory DNA methylation patterns and histone acetylation-related pathways	Reduced inflammation, improved insulin sensitivity, improved lipid profile	[66,69]
DASH diet	DNA methylation and chromatin-related changes associated with blood pressure regulation	Reduced blood pressure, improved endothelial function	[71]
Western diet	Hypomethylation of inflammatory genes (TNF-α, IL1B), altered methylation of metabolic loci	Increased inflammation, obesity risk, insulin resistance	[68,76]
Vegetarian diet	Global DNA methylation changes; modulation of longevity-related genes	Reduced epigenetic aging markers, improved oxidative stress profile	[72]
Caloric restriction	Activation of SIRT1 and AMPK pathways, modulation of miRNA expression	Enhanced mitochondrial function, reduced hepatic steatosis, improved metabolic flexibility	[13,48,67]

## Data Availability

No new data were created or analyzed in this study. Data sharing is not applicable to this study.
